# Zinc: From Biological Functions to Therapeutic Potential

**DOI:** 10.3390/ijms24054822

**Published:** 2023-03-02

**Authors:** Maria Inês Costa, Ana Bela Sarmento-Ribeiro, Ana Cristina Gonçalves

**Affiliations:** 1 Laboratory of Oncobiology and Hematology (LOH), University Clinics of Hematology and Oncology, Faculty of Medicine (FMUC), University of Coimbra, 3000-548 Coimbra, Portugal; 2 Coimbra Institute for Clinical and Biomedical Research (iCBR)—Group of Environmental Genetics of Oncobiology (CIMAGO), Faculty of Medicine (FMUC), University of Coimbra, 3000-548 Coimbra, Portugal; 3 Center for Innovative Biomedicine and Biotechnology (CIBB), 3004-504 Coimbra, Portugal; 4 Clinical Academic Center of Coimbra (CACC), 3000-061 Coimbra, Portugal; 5 Hematology Service, Centro Hospitalar e Universitário de Coimbra (CHUC), 3000-061 Coimbra, Portugal

**Keywords:** zinc signals, zinc transporters, zinc signaling, cell proliferation, cell death, DNA repair, therapeutic target

## Abstract

The trace element zinc (Zn) displays a wide range of biological functions. Zn ions control intercellular communication and intracellular events that maintain normal physiological processes. These effects are achieved through the modulation of several Zn-dependent proteins, including transcription factors and enzymes of key cell signaling pathways, namely those involved in proliferation, apoptosis, and antioxidant defenses. Efficient homeostatic systems carefully regulate intracellular Zn concentrations. However, perturbed Zn homeostasis has been implicated in the pathogenesis of several chronic human diseases, such as cancer, diabetes, depression, Wilson’s disease, Alzheimer’s disease, and other age-related diseases. This review focuses on Zn’s roles in cell proliferation, survival/death, and DNA repair mechanisms, outlines some biological Zn targets, and addresses the therapeutic potential of Zn supplementation in some human diseases.

## 1. Introduction

Zinc (Zn) is one of the most abundant micronutrients in the human body, known for its role as the regulatory, structural, and catalytic component of at least 3000 proteins, including enzymes and transcription factors [1]. More recently, the role of Zn as a regulatory and signaling element in intercellular and intracellular communication has also been acknowledged [2,3]. The indispensability of Zn in cell biology is reflected by the fact that approximately 10% of the human proteome is associated with Zn ions for regulation of gene expression, DNA metabolism, chromatin structure, cell proliferation, maturation, death, immune responses, and antioxidant defenses [1].

Given the broad spectrum of functions that require Zn, levels within the cell are tightly regulated to maintain narrow physiological concentrations. In contrast to other metals (e.g., iron, copper, and mercury) that may accumulate excessively in the organism and reach levels of toxicity, Zn uptake, subcellular distribution, storage, and excretion are efficiently controlled. The major players involved in Zn homeostasis are the Zn importers Zrt- and Irt-like proteins (ZIP), Zn transporters (ZnT), and metallothioneins (MT) [1,4,5].

**Figure 1:**
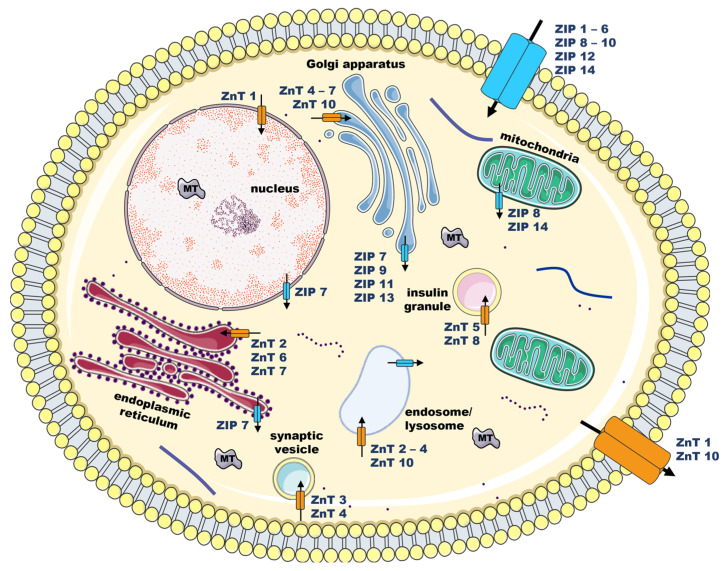
Schematic illustration of zinc (Zn) homeostasis mechanisms and potential subcellular locations of Zn channels. Regulation of cellular zinc fluxes in and out of the cell and cellular compartments is controlled by several Zrt- and Irt-like proteins (ZIP; blue)—the importers—and Zn transporters (ZnT; orange)—the exporters. Metallothioneins (MT; grey) are also key proteins in Zn homeostasis; MT act as Zn buffers by binding to these ions and releasing them into the cytoplasm to increase free intracellular Zn pools, according to cellular needs. Arrows indicate the direction of Zn mobilization through Zn channels. (Figure created with Servier Medical Art and Biorender).

ZIP and ZnT regulate Zn fluxes across the plasma membrane and through cellular compartments (Figure 1). ZIP increases cytosolic Zn levels by allowing Zn uptake from the extracellular environment or efflux from intracellular organelles to the cytoplasm. Intracellularly, Zn is mostly bound to small cysteine-rich proteins, MT, that maintain free Zn at low levels. MT regulates Zn bioavailability by sequestering, mobilizing, and releasing Zn ions in response to various stimuli [1,6]. The exporters, ZnT, carry Zn out of the cell or into intracellular storage compartments, thus reducing the concentration of cytosolic Zn [1,4,5]. When intracellular Zn exceeds its upper limit, a regulatory loop dependent on the activation of the Zn sensor metal response element-binding transcription factor-1 (MTF-1) induces the transcriptional expression of MT and some ZnT to buffer and export excess of Zn from the cell [1,7]. By maintaining Zn homeostasis, Zn channels and MT regulate Zn-dependent proteins, which link impaired expression and/or activity of ZIP, ZnT, and MT to the onset and progression of various human disorders [7].

Until recently, the extent of Zn’s biological implications has been underestimated. However, increasing evidence shows that this trace element regulates major cellular events in a variety of ways. This review focuses on important aspects of Zn in cell biology, particularly cell–cell communication and cell signaling, proliferation, survival, and DNA repair. The potential of Zn as a therapeutic agent in several human diseases is also addressed.

## 2. Zinc (Zn) and Cell Signaling

### 2.1. Extracellular Zn Targets

The structural and catalytic functions of Zn have long been known, but the role of this micronutrient in cell signaling is a more recent discovery. The detection of Zn ions in presynaptic vesicles of specialized neurons and their release upon exocytic stimulation led to the proposal of Zn as the carrier of information between cells. Similar to neurotransmitters, Zn is stored and released from synaptic vesicles and is taken up by postsynaptic cells, mediating intercellular communication [8,9,10]. After vesicular exocytosis, Zn can act as the first messenger, interacting with plasma membrane receptors to generate intracellular responses. Moreover, cells can also take up Zn via transmitter-gated ion channels and modulate intracellular targets [8,9,10].

The involvement of Zn in neuronal communication encouraged studies in other cell types. Secreting cells such as those of the prostate, mammary glands, endocrine and exocrine pancreas, intestine, immune system, and platelets contain a remarkable Zn content within their vesicles [2,11]. In these cells, Zn supports synthesis and secretion functions and is released through vesicular exocytosis for the paracrine, autocrine, and endocrine modulation of multiple targets [2,12,13]. Extracellular targets of Zn signals (sensors or receptors) remain, in general, largely uncharacterized, but some have been identified. In neuronal communication, Zn released into the synapse can bind to neuronal receptors (e.g., N-methyl-D-aspartate (NMDA), gamma-aminobutyric acid (GABA), glycine receptors, L-type and N-type voltage-gated calcium channels (VGCCs), voltage-gated potassium channels, P2X purinergic receptors, and transient receptor potential ankyrin 1 (TRPA1) channels) to mediate excitatory or inhibitory responses [8,9,10]. Zn shows variable modulatory effects on these targets, depending on the targets’ isoforms, Zn concentration, and synaptic activity [10].

Extracellular Zn also activates growth factor receptors, such as the epidermal growth factor (EGF) and insulin growth factor (IGF) receptors, which control cell proliferation, growth, and survival pathways [11]. Another distinct target for extracellular Zn is the ZnR/GPR39 coupled Zn receptor with G protein. ZnR/GPR39 is expressed in neurons, colonocytes, keratinocytes, and pancreatic, prostate, salivary gland, and thyroid cells, among other tissues in which Zn signaling appears to be relevant [12,14,15,16]. The first recognized functional outcome of ZnR/GPR39 activation is the stimulation of intracellular calcium (Ca^2+^) signaling [14,16]. Bound to the receptor, extracellular Zn triggers Ca^2+^ release from intracellular storages through inositol triphosphate (IP3) activation, which ultimately leads to the modulation of intracellular phosphorylation pathways. The mitogen-activated protein kinase (MAPK) and the phosphoinositide 3-kinase (PI3K)/protein kinase B (AKT) pathways are the most widely induced by ZnR/GPR39 activation and show essential functions in proliferation and survival, especially in epithelial cells [12,14,16,17]. In neurons, signals generated secondarily to ZnR/GPR39 activation are also important for synaptic plasticity, learning, and memory [8,12].

In addition to Ca^2+^ regulation downstream of ZnR/GPR39 activation, other ion transporter mechanisms can be regulated (e.g., sodium (Na^+^), potassium (K^+^), and chlorine (Cl^−^) channels) [14]. Such elements have important functions in epithelial and neuronal cells. For instance, the Na^+^/hydrogen antiporter (Na^+^/H^+^ exchanger), upregulated by ZnR/GPR39 activation, controls intracellular pH in colonocytes, keratinocytes, and neurons [12,14,17]. This constitutes a key Zn-dependent mechanism for maintaining pH homeostasis, which is essential for cell survival, migration, the permeability barrier, and neuronal excitability [12,14]. The K^+^/Cl^−^ cotransporters, required for the regulation of cell volume, ion transport, and neuronal excitability, are also modulated downstream of ZnR/GPR39 signaling, either through phosphorylation reactions or by changes in expression [12,14,18]. These examples illustrate how a single receptor activated in response to transient changes in extracellular Zn mediates several Zn-dependent responses. Given the multitude of Zn-interacting proteins in the human proteome, it is expected that virtually any of these ligands may play a role in signaling transduction, generating a wide range of Zn-related responses that remain to be identified.

### 2.2. Intracellular Zn Signaling

Extracellular Zn modulates the bioavailability of several ligands and the binding affinity of their corresponding receptors, thereby influencing the initiation of intracellular signaling pathways. Moreover, in addition to their extracellular functions, cell biology studies indicate the importance of intracellular Zn in cell signaling. Intracellularly, Zn modulates signaling pathways at many levels. For instance, by interfering with the activity of proteins such as MAPK, Ca^2+^/calmodulin-activated protein kinase-2 (CaMPK-2), protein kinase C (PKC), P70S6 kinase (P70S6K), cyclic nucleotide phosphodiesterases (PDE), and protein tyrosine phosphatases (PTP), Zn suppresses phosphatase activity and promotes phosphorylation reactions [2,3,6,19,20,21].

Initially, extracellular Zn taken up by cells was thought to be the main source of intracellular Zn signaling. For example, in neurons, the presynaptic cell recaptures Zn released into the synapse to regulate phosphorylation signaling [2]. However, observations in mast cells of Zn waves arising from intracellular storages, to which extracellular Zn does not contribute, strengthened the hypothesis that intracellular Zn reservoirs modulate responses to external conditions [22]. Subsequent studies on other cell types confirmed the presence of Zn transporters in organelles such as the endoplasmic reticulum (ER), Golgi apparatus, and mitochondria and demonstrated its involvement in the regulation of Zn fluxes for intracellular signaling [8,12]. Although most reports identify ZIP and ZnT as the main regulators of these fluxes, other ion transporters and channels may be implicated. Outside intracellular compartments, the increase in cytosolic Zn in response to extracellular stimuli mainly depends on its release from MT [11].

**Figure 2:**
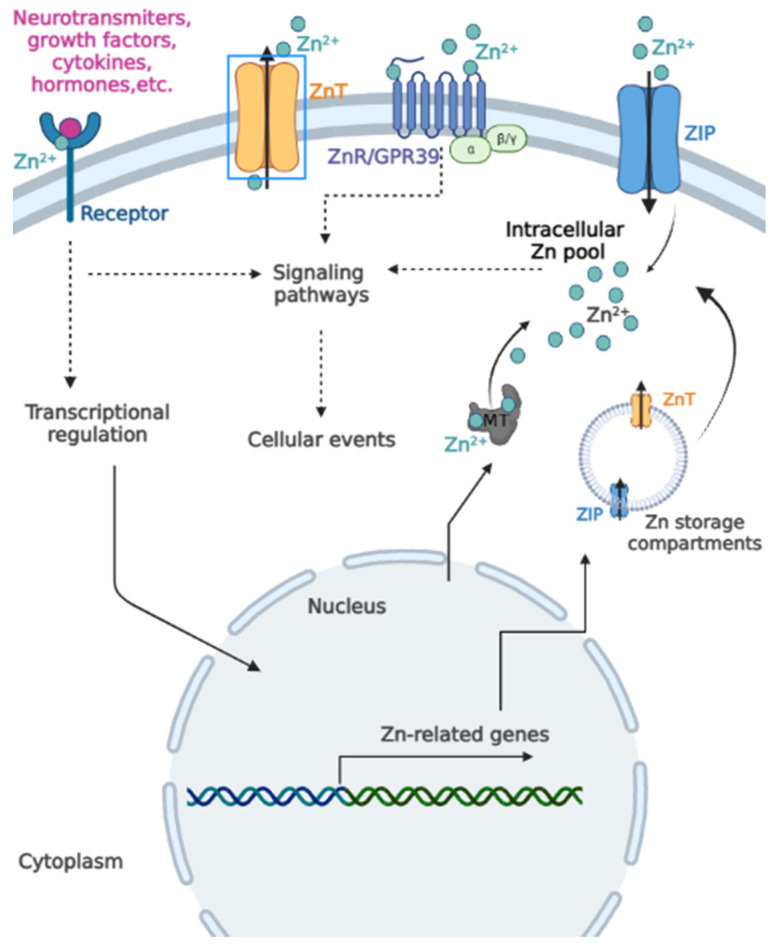
Integration of extracellular and intracellular Zn signals and the labile Zn pool. Zn can increase the affinity of several ligands (e.g., neurotransmitters, growth factors, cytokines, hormones, among others) to their corresponding receptors, thus promoting the initiation of intracellular signaling pathways. These extracellular stimuli can also directly or indirectly affect intracellular Zn status by modulating the transcriptional regulation of Zn-related genes. Such interactions generate several Zn-related responses, such as modulation of Zn transporters and release of Zn from intracellular compartments and MT, all of which increase the intracellular Zn pool. The intracellular labile Zn serves various functions by modulating Zn-dependent proteins from different signaling cascades related to cellular events such as proliferation, growth, survival, and apoptosis, among others. Extracellular Zn can also directly bind to Zn receptors such as ZnR/GPR39 and initiate intracellular signaling cascades. (Figure created with Biorender).

Despite the fact that most intracellular Zn is tightly bound to high-affinity Zn-binding proteins such as MT and glutathione (GSH), there is a small pool of free Zn, oscillating from picomolar to low nanomolar concentrations, with central functions as a second messenger in intracellular communication [6,11,21]. For instance, estimates of labile zinc concentrations are 24 pmol/L in erythrocytes, 0.17–2.5 nM in peripheral venous blood monocytes, and 0.35–4.4 nM in lymphocytes, depending on the quantification method [23,24]. Evaluation of free Zn in live HeLa cells also showed variations in different subcellular compartments (i.e., ER—14 pM; mitochondria—60 pM; nucleus—0.11 nM, cytosol—0.13 nM, Golgi apparatus—25 nM) [25]. Since fluctuations within this range match the binding affinity of several molecules relevant to signal transduction, it is proposed that labile Zn is a potent modulator of different signaling pathways and a mediator of diverse cellular responses (Figure 2) [26]. Similar to other second messengers, intracellular Zn captures signals from cell surface receptors (e.g., those activated by growth factors, hormones, and cytokines) and transfers the external stimuli to cytoplasmic or nuclear targets to generate biological responses. Thus, fluctuations in the labile Zn content can significantly alter cells’ activities. Such oscillations can be induced by electrical stimulation of excitable cells, mitochondrial release of Zn, cellular Zn influxes, exposure to DNA-damaging agents, and oxidative release of Zn from proteins [26].

Three main types of intracellular Zn signals have been characterized according to their nature and duration. Zn fluxes and Zn waves are transcriptionally independent Zn signaling events and promote rapid changes in cytosolic Zn levels [11]. These represent rapid mechanisms of Zn release from intracellular compartments and occur as early responses to extracellular sources, in which Zn acts as a transducer of extracellular stimuli into intracellular events [11,19]. Zn fluxes and Zn waves can be distinguished mainly due to their duration: the first does not last long, whereas the latter continuously increases for at least an hour after induction and is largely associated with mast cells. A third type of Zn signal, dependent on the transcriptional regulation of Zn transporters, is also considered. This represents a late, nonimmediate response in which intracellular Zn levels are altered several hours after the external stimuli due to changes in transporters’ expression. This signal can last for one or more days [11,19].

Thus, Zn is involved in extracellular signal recognition, signal transduction, and second messenger metabolism [6,21]. The list of known second messengers includes Ca^2+^, cyclic adenosine monophosphate (cAMP), cyclic guanosine monophosphate (cGMP), G proteins, protein kinases, protein phosphatases, nitric oxide (NO), lipid mediators, and nuclear receptors [19]. More than just a messenger itself, Zn is also a regulator of other second messengers. For example, the regulation of free intracellular Ca^2+^ depends on Zn. As stated in Section 2.1, activation of the ZnR/GPR39 receptor by extracellular Zn induces intracellular Ca^2+^ mobilization. However, in addition to extracellular Zn regulating Ca^2+^ gradients, intracellular Zn pools also affect Ca^2+^ mobilization [3,12,27]. In cardiomyocytes, increases in cytosolic Zn released from the ER also regulate Ca^2+^ efflux from these reservoirs [12,27]. This ability of Zn to alter Ca^2+^ dynamics in cardiac tissue may partly explain the contribution of perturbed Zn homeostasis to cardiac dysfunctions (e.g., altered contractility and heart failure) [27].

Interestingly, there seems to be a bidirectional interaction between Zn and Ca^2+^ signaling. While Zn signals frequently involve a downstream modulation of Ca^2+^, Ca^2+^ gradients can also arise upstream of the Zn signal. For example, the release of Zn from cellular vesicles to the extracellular environment is mediated by Ca^2+^-dependent exocytosis [2,13]. Studies on cardiac excitation found that intracellular Ca^2+^ pools regulate Zn homeostasis in cardiomyocytes, with increases in free intracellular Ca^2+^ triggering Zn release and reactive oxygen species (ROS) production during the cardiac cycle [28]. Furthermore, the entry of extracellular Ca^2+^ into mast cells is also required to generate Zn waves from the ER [16,19]. Under oxidative stress conditions, Ca^2+^/calmodulin also has an upstream role in Zn signaling, generating a NO signal that targets MT for Zn release [2,8]. In such conditions, Zn initiates antioxidant and repair responses to restore cellular balance [2]. The activity of CaMPK-2, a serine/threonine-specific kinase involved in many signaling pathways and a major neuronal kinase, is also modulated by Zn in a concentration-dependent manner. Low Zn concentrations are reported to stimulate kinase activity independently of Ca^2+^/calmodulin, whereas high Zn levels inhibit the binding of Ca^2+^/calmodulin and inactivate the substrate phosphorylation activity of CaMPK-2 [3,29].

Zn also influences the cellular content of the second messengers cAMP and cGMP, thus influencing cyclic nucleotide signaling [3,21,30,31]. Levels of cAMP and cGMP are regulated through synthesis by adenylate and guanylate cyclases and degradation by the cyclic nucleotide PDE [3,31]. The catalytic domain of some PDE isoforms contains Zn-binding motifs, which means that Zn can directly and reversibly affect PDE function [3,31]. Low concentrations of Zn are essential for PDE activity, while higher concentrations demonstrate clear inhibitory effects [3]. The stimulatory or inhibitory effects of Zn on PDE activity affect a multitude of signaling pathways for which cAMP and cGMP are required. This is highlighted, for instance, in immunity studies showing that Zn interferes with cytokine production by monocytes through modulation of PDE enzymes and cyclic nucleotide signaling [31]. In such studies, high Zn concentrations repressed the lipopolysaccharide (LPS)-induced activity of several PDE isoforms (e.g., PDE-1, PDE-2, and PDE-4) in monocytes, increasing cGMP levels. This, in turn, suppressed nuclear factor kappa-light-chain-enhancer of activated B cells (NF-kB) signaling and the subsequent release of tumor necrosis factor-alpha (TNF-α) and interleukin-1 beta (IL-1β) [31]. Interestingly, not only was PDE activity reduced by Zn but the expression of PDE genes was also repressed by a high intracellular Zn content [31]. Such results indicate the role of Zn in immunomodulation through PDE-mediated suppression of proinflammatory cytokines.

PKC is also Zn-dependent for structural and catalytic purposes since most isoforms contain Zn-binding motifs in their regulatory region and require nanomolar Zn concentrations for enzymatic function [1]. Studies have shown considerable plasticity of Zn ions within the Zn-finger structure of PKC, which correlates to enzyme function. Specifically, the main enzyme activators (i.e., binding of lipid second messengers and ROS) converge at the Zn finger and trigger Zn release, a crucial step for the initiation of PKC catalytic function [3,32]. This activating role is also supported by observations in neuronal cultures of mice, in which short-term exposure to micromolar concentrations of Zn increased PKC activity, resulting in Zn-induced cortical neuronal cell death [33].

These examples show the ability of Zn to modulate phosphorylation signaling, which is a key feature of this trace element in cell biology. The activating or inhibitory interactions that Zn establishes with several enzymes alter the phosphorylation state of proteins and transcription factors, thereby influencing gene expression and biological outcomes. In the following subsections, we discuss the involvement of Zn in relevant intracellular biological pathways and identify some Zn targets in these signaling cascades.

## 3. Zn and Cell Proliferation

Cell proliferation is highly regulated by a plethora of mitogenic and antiproliferative signals that converge on cell cycle machinery, led by the activators cyclins and cyclin-dependent kinases (CDK) and restricted by CDK inhibitors [34]. The requirement of Zn for cell proliferation is supported by its structural and catalytic functions in enzymes related to DNA synthesis, transcription, and ribosomal function, as well as its role as a mitogenic signal messenger [3,35]. 

Zn can directly or indirectly enhance mitogenic signaling pathways by modulating growth factors and their receptors, the structure and function of enzymes, as well as phosphorylation reactions and downstream events [36]. Kinase and phosphatase activities, deeply involved in the regulation of cell division and proliferation, are strongly modulated by Zn. Experiments in rat glioma cells (C6 cell line) indicated that protein tyrosine phosphorylation and mitogenic stimuli were enhanced by increases in extracellular Zn, and significantly reduced by Zn depletion. Moreover, the authors found that the stimulation of phosphotyrosine signaling occurred concomitantly to the inhibition of PTP activity and stimulation of insulin and IGF-1 signaling [36]. Therefore, the stimulatory effects of Zn on mitogenic signaling can be the result of at least three properties: (1) its role in the hormonal regulation of cell proliferation, (2) its activating effect on protein phosphorylation, and (3) its suppressor effect on phosphatase activity, which allows the maintenance of phosphorylation signaling [36].

Growth factors, cytokines, and hormones are primary inducers of cell proliferation [3,35,37]. These molecules activate MAPK, PI3K/AKT, and PKC signaling, all involved in cell growth and proliferation [20,37,38,39,40]. The phosphorylation reactions of the MAPK cascade, activated by growth factors (e.g., IGF-1 and IGF-2), culminate in the nuclear translocation of extracellular signal-regulated kinases (ERK) and the activation of transcription factors that control key cell cycle events. Different studies have shown the effects of Zn depletion and supplementation on mitogenic signaling. Zinc deficiency was shown to reduce IGF-1 levels both in humans and rats [41,42]. Accordingly, in murine fibroblasts, increases in extracellular Zn improved the affinity of IGF-1 and IGF-2 to the IGF receptor and enhanced protein tyrosine phosphorylation and MAPK signaling, leading to cell proliferation [39,43]. On the other hand, Zn chelation abolished MAPK activity in rat fibroblasts, which was reversed by Zn addition [44]. In human bronchial epithelial cells, Zn also induced EGF phosphorylation and MAPK activity [45]. As MAPK inducers, these Zn-related interactions mediate G_1_/S transitions and stimulate proliferation, whereas Zn deficiency has been shown to consistently reduce IGF-1 levels and MAPK activity in both animal models and humans, resulting in proliferation inhibition [3,37]. The secretion of growth hormone (GH) from the pituitary gland also decreases in low-Zn conditions, as observed by low GH expression in the rat liver under Zn depletion [46]. Since GH stimulates liver synthesis and the secretion of IGF-1, this decrease correlates with the low levels of IGF-1 observed in Zn deficiency and the consequent decrease in cell proliferation [39]. Regulation of cyclin D1 expression, together with CDK4/6, is also of great relevance. These proteins mediate G_1_/S transitions, a point of no return in the cell cycle which commits cells to complete division [34,37]. In a human neuroblastoma cell line (IMR-32) and primary cultures of rat cortical neurons, inhibition of proliferation by low Zn was associated with hypophosphorylation of ERK, downregulation of cyclin D1, and arrest of G_0_/G_1_ [37]. 

Although the MAPK pathway is one of the best characterized regarding cell cycle control, other signals also regulate proliferation. These include PI3K/AKT, PKC, and NF-kB [34]. Similar to MAPK, the stimulation of PI3K/AKT and PKC can occur as a result of IGF receptor activation. Activation of these pathways by Zn has been observed in several cell types and animal models. For example, studies in myogenic cells revealed that Zn and insulin synergize to promote AKT phosphorylation and cell proliferation [40]. Similar observations have been reported in mice adipocytes and fibroblasts [47]. PKC activity is also driven by Zn in a Ca^2+^-dependent manner, demonstrating that Zn and Ca^2+^ can synergize to integrate mitogenic stimuli [39,48].

Another kinase involved in cell cycle progression is thymidine kinase (TK), whose activation increases notably during the G_1_ and S phases [29]. This enzyme catalyzes the phosphorylation of deoxythymidine to deoxythymidine monophosphate, which is required for DNA synthesis and cell division [39]. Although TK is not a Zn metalloenzyme, it requires Zn for its transcriptional regulation, stimulating mRNA synthesis by binding Zn-dependent proteins to the promoter region of the thymidine kinase gene (*TK1*). Low-Zn conditions correlate with decreased TK levels and inhibition of DNA synthesis, while Zn addition reverses these effects [39]. This has been demonstrated in in vitro Zn chelation studies and in animal models fed with Zn-deficient diets, both leading to decreased thymidine incorporation in the DNA, reduced TK activity, and low TK mRNA concentration [49,50].

Other proliferation-related Zn targets have been studied, although a clear regulatory effect has not been established. This is the case for the transcription factor complex NF-kB. In addition to its well-known roles in inflammation and immune response, NF-kB is also highly involved in cell proliferation and differentiation [51,52]. Activated by various environmental stressors, cytokines, and growth factors, NF-kB translocates to the nucleus and regulates genes related to cell growth, proliferation, differentiation, apoptosis, inflammation, and immune function [51,53]. NF-kB does not contain Zn in its structure, but the ability of this transcription factor to bind to its DNA targets seems to be influenced by Zn. However, reports include both inhibitory and stimulatory effects, and there is no clear correlation between Zn deficiency and the expression of NF-kB-dependent genes [21,30,51,53]. While some studies indicate an increase in the DNA-binding activity of NF-kB under low-Zn conditions, others suggest that the nuclear translocation of active NF-kB is compromised by Zn deficiency, leading to a downregulation of NF-kB targets and inhibition of proliferation and survival pathways [21,51]. Examples of such are the reports of Bao et al. which indicate that Zn addition increases the translocation and activation of NF-kB via IkB phosphorylation in HUT-78 (Th_0_) cells [54]. Others, such as as Ho and coworkers, suggested that Zn supplementation inhibits NF-kB and downregulates its downstream targets in a mouse model of diabetes [55]. Since no consensual results have been reached and results vary substantially between models, it has been proposed that the effects of Zn on NF-kB signaling may be cell- and context-specific [51,53]. 

**Figure 3:**
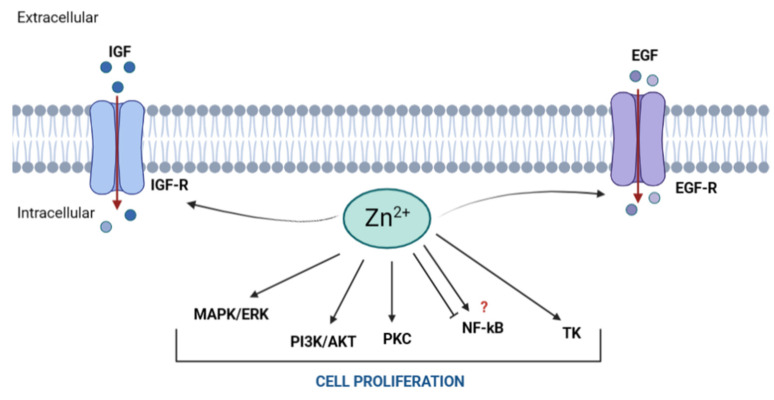
Targets of Zn in cell proliferation. Zn increases the affinity of insulin growth factor (IGF) and epidermal growth factor (EGF) for its receptors. Activation of IGF and EGF initiates several signaling cascades, such as MAPK/ERK, PI3K/AKT, and PKC, to stimulate cell growth and proliferation. The role of Zn in the modulation of NF-kB is still controversial since both activator and inhibitory roles have been described. Lastly, Zn also plays a role in the promotion of thymidine kinase (TK) mRNA synthesis, a key enzyme for cell cycle progression. The red question mark (?) represents the nonconsensual effects of Zn on NF-kB signaling. (Figure created with Biorender).

The fact that Zn can control cell entry into the S phase through several targets and modulate the activity of numerous enzymes required for DNA synthesis demonstrates its strong involvement in cell proliferation. Other findings reinforce this biological role. Tubulin polymerization, for instance, seems to involve Zn. Impaired tubulin polymerization is observed under low-Zn conditions, affecting microtubule assembly and the formation of the mitotic spindle required for mitosis [37]. Changes in MT also validate the stimulatory role of Zn in cell proliferation. MT expression is commonly higher in proliferating tissues (e.g., regenerating and developing rat liver, tumor cells) compared to nonproliferating cells [56,57,58]. Moreover, MT levels oscillate during cell cycle stages. Interestingly, studies indicate that in such highly proliferating tissues, the maximum peak of MT is reached in the G_1_/S transitions, suggesting a role in the stimulation of cancer cell proliferation [59]. Moreover, not only do cellular levels of MT vary, but their subcellular location also differs depending on the cell cycle phase. While the majority of MT content is normally found in the cytoplasm, transient increases in the nuclear MT fraction during cell division have been observed in primary rat hepatocytes, regenerating rat liver, and certain tumors [58,60,61,62]. The increased expression of MT and its nuclear translocation has been proposed as a means by which cells increase nuclear Zn for mitogenic purposes [3,63]. The main interactions of Zn with molecular targets related to cell proliferation are represented in Figure 3.

## 4. Zn and Cell Death

An adequate balance between the forces that drive cell division and death is of utmost importance for the maintenance of general homeostasis. Zn ions influence proliferation and apoptosis by coordinating the mechanisms involved in such opposing events [64,65]. Consistent with its roles in mitogenic and survival signaling, Zn is commonly considered a suppressor of apoptosis [37,65]. In fact, most studies emphasize the antiapoptotic functions of Zn, with Zn depletion experiments extensively reporting proapoptotic effects in several cell types (e.g., normal fibroblasts, hepatocytes, precursors of T cells, and testicular cells) [35]. For example, Zn deficiency reduces neuronal cell viability, which can be accompanied by changes in neuronal amino acid neurotransmitter content and altered glutamate receptor expression [66]. HeLa cells treated with Zn chelators also displayed an increased apoptotic rate, through the activation of caspases-3, -8, and -9 and cleavage of caspase targets (e.g., Poly(ADP-ribose) polymerase (PARP) proteins) [67]. A study by Lee and collaborators also found that Zn depletion led to apoptosis in osteoblasts, accompanied by the induction of the Janus kinase 2 (JAK2)–signal transducer and activator of transcription 3 (STAT3) pathway [68]. Mice fed with a Zn-deficient diet also showed increased testicular cell apoptosis, associated with severe oxidative stress, mitochondrial damage, and altered MAPK and NF-kB signaling, reflecting the role of Zn in normal spermatogenesis [69]. On the other hand, Zn supplementation interventions designed to correct Zn deficiencies are shown to prevent apoptosis in the presence of apoptogenic agents, while low-Zn conditions enhance their apoptotic effect. Hao et al. found that Zn addition prevented cell death induced by uranyl nitrate exposure in a human kidney proximal tubular cell line (HK-2) by multiple independent mechanisms (e.g., reduction of oxidative stress, maintenance of mitochondrial membrane potential, inhibition of caspase activation, induction of MT expression, and modulation of proapoptotic and antiapoptotic proteins) [70]. In the human keratinocyte cell line HaCaT, Zn also prevented the apoptogenic effects of ropivacaine [71]. Similarly, Zn supplementation protected TM3 Leydig cells from heat stress-induced apoptosis, via ER stress inhibition [72]. 

However, Zn may also exhibit proapoptotic effects, especially when the intracellular concentration surpasses the capacity for homeostatic control and Zn buffering is compromised [73]. Intracellular Zn pools are so closely involved in cell death regulation that small variations in labile Zn content can significantly alter the susceptibility of cells to apoptosis [65]. When Zn levels exceed the upper limit, mitochondrial membrane permeability is compromised, resulting in cytochrome *c* release and inhibition of respiratory chain reactions [30]. Consequently, cell death occurs secondarily due to damage induced by altered redox homeostasis and deleterious interactions with biomolecules [3,73]. In this context, cell-buffering capacity appears to be a major decisive factor in cell fate. The state of activity of molecules responsible for Zn homeostasis (i.e., ZIP, ZnT, and MT) may be one of the most crucial factors establishing the threshold between antiapoptotic and proapoptotic effects [73]. 

**Figure 4:**
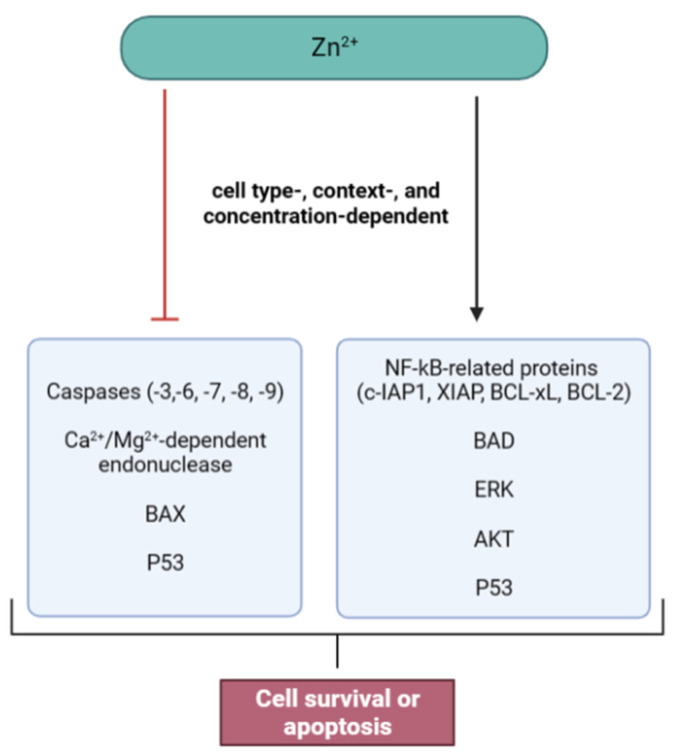
The effects of Zn on the regulation of cell death and survival mechanisms. Most studies show that under physiological conditions, Zn exhibits antiapoptotic functions. However, reports of proapoptotic effects of Zn have also been described. The effects of Zn on cell death and survival pathways seem to depend on cell type, context, and concentration. The enzymatic function of proapoptotic proteins such as caspases, Ca^2+^/Mg^2+^-dependent endonuclease, and BAX are normally inactivated by Zn. However, antiapoptotic proteins, such as NF-kB-induced survival molecules, BAD, and growth, proliferation, and survival regulators, such as ERK and AKT, are usually activated by Zn. Taken together, these effects usually result in the prevention of apoptosis. However, given that other conditions may influence Zn effects, it is possible that Zn may also regulate these targets to promote proapoptotic effects. The role of Zn in modulating P53 activity is still controversial, as both activator and inhibitory roles have been described. (Figure created with Biorender)

The anti-/proapoptotic effects of Zn are also cell type- and context-dependent. In fact, despite generally showing antiapoptotic effects under normal conditions, Zn can primarily trigger apoptosis in specific cells [74,75]. In neurons, for example, after releasing into the synapse, the rapid influx of Zn through postsynaptic channels generates highly neurotoxic ROS and stimulates cell death [11]. However, as previously discussed, Zn deficiency has also been associated with apoptosis induction in proliferating and quiescent neuronal cells. A factor of consideration when taking into account these inconsistent effects is the conditions under which laboratorial experiments are performed, especially when using cellular models. It can be pointed that Zn depletion studies are more reliable in the definition of Zn effects than supplementation experiments, since the latter usually employ concentrations much higher than what is physiologically relevant. However, the complete depletion of Zn also does not accurately mimic physiological or pathological conditions [65,75]. Cell context also influences Zn effects, with many reports stating proapoptotic effects of Zn in malignant cells (e.g., ovarian cancer, pancreatic adenocarcinoma, and prostatic cancer cells) in opposition to antiapoptotic effects in normal cells [76,77,78,79]. Therefore, the effects may depend on several internal and external factors, and careful extrapolations need to be made when analyzing such results. Despite the gaps that are yet to be filled, it is undeniable that Zn interacts with a wide range of nuclear and cytoplasmic molecules of apoptotic pathways, as well as some found in mitochondria and microtubules [65]. Throughout the years, studies have allowed the characterization of some targets, although the exact regulatory mechanisms still need further investigation (Figure 4).

The decision to undergo apoptosis requires a set of cellular events, such as cell cycle arrest and induction of apoptotic cascades. Zn stimulates proliferation and prosurvival molecules such as ERK, AKT, and NF-kB, and modulates apoptosis regulators such as tumor protein 53 (P53), caspases, and antiapoptotic and proapoptotic proteins of the B-cell leukemia/lymphoma 2 (BCL-2) family [37,80]. Altered tyrosine kinase signaling, decreased stimulation of survival pathways, mitochondrial dysfunction, and changes in caspase regulation underlie Zn deficiency-induced apoptosis [35,37,80]. Accordingly, in neuronal cells, low-Zn conditions have been related to ERK and AKT hypophosphorylation, downregulation of NF-kB-dependent prosurvival genes, and caspase-3 activation [37]. P53 activation, mitochondrial alterations (e.g., increase in BCL-2 associated X protein (BAX), translocation of the apoptosis-inducing factor (AIF) to the nucleus, changes in mitochondrial membrane potential, and ROS production), and increases in caspases-2, -3, -6, and -7 mRNA concentrations and protein activity were also reported in human neuronal cell lines [80].

Interestingly, proliferation and survival pathways are so deeply intertwined that the disruption of growth factors’ signal transducer pathways mediated by receptor tyrosine kinases disturbs cell proliferation and induces apoptosis [37,64]. In fact, decreased growth factor signaling and the disruption of proliferation machinery precede the induction of cell death [64]. Zn deficiency results in the deactivation of growth factor pathways. Importantly, disruption of these signaling cascades can occur due to the withdrawal of growth factors in the absence of Zn or downstream of ligand binding due to defects in the signaling of receptor tyrosine kinases [64]. By limiting the kinase activities of ERK and AKT, Zn deficiency induces cell cycle arrest, limits proliferation, and promotes cell death [64]. For example, phosphorylation of BCL-2-associated death promoter (BAD), a proapoptotic protein, by these kinases is fundamental for cell signaling inactivation and survival [37]. When phosphorylated in at least three serine residues, BAD remains in the cytoplasm and its translocation to the mitochondria, where it would stimulate the release of cytochrome *c* and the activation of caspase-3, is prevented [37]. In rat cortical neurons, Erk inhibition under low-Zn conditions correlates with changes in BAD phosphorylation and the induction of apoptosis [37]. Furthermore, reports from Zn supplementation studies indicate increased ratios of BCL-2/BAX. For example, in a human premonocytic cell line (U937), prevention of hydrogen peroxide (H_2_O_2_)-induced apoptosis by Zn addition was mediated by increases in the BCL-2/BAX ratio [81]. Considering that BCL-2 is an antiapoptotic protein and BAX is an inducer of apoptosis, this supports the idea that Zn increases cell survival and decreases susceptibility to apoptosis. The requirement of AKT and ERK for BAD inactivation represents a survival mechanism supported by growth factors that depend on Zn, providing a key link between proliferation, survival, and this trace element. Additionally, Zn regulates cell death by other targets, namely NF-kB. Most Zn chelation assays report decreased transcription rates of NF-kB-dependent genes encoding antiapoptotic proteins (e.g., cellular inhibitor of apoptosis protein 1 (c-IAP1), X-linked inhibitor of apoptosis protein (XIAP), B-cell lymphoma-extra-large protein (BCL-xL), and BCL-2) [37]. Despite the nonconsensual roles of Zn in NF-kB modulation, there seems to be decreased NF-kB-dependent antiapoptotic signaling in Zn deficiency. It is important to note that reduced NF-kB-induced transcription under low-Zn conditions may also be a consequence of a less active RNA polymerase II, which contains seven Zn-binding sites in its structure. Thus, the interactions of Zn with a broad selection of targets may have major contributions to Zn-induced apoptotic effects.

Given the central regulatory role of P53 for several biological functions, the interaction of Zn with this protein requires special attention. The exact mechanisms underlying apoptosis induction are unclear, but DNA damage and the subsequent activation of P53 are well-known initial triggers [64,82]. Upon DNA damage and other stress signals, P53 accumulates in the nucleus, binds to specific DNA regions, and regulates the expression of effector genes related to antiproliferative responses, DNA repair, apoptosis, and senescence [83,84]. The DNA-binding domain of P53 is stabilized by Zn and P53’s dependence on Zn for stability and function is demonstrated by Zn chelation experiments consistently reporting P53 denaturation, with subsequent supplementation restoring the native wild-type form [83,84]. Thus, even though P53 upregulation is often found in Zn deficiency, it is often accompanied by dysfunctional protein activity [64]. Therefore, despite the fact that low Zn levels often correlate with cell death, the role of P53 under such conditions is not consensual. A study on rat glioma cells (C6 cell line) elucidates this, showing that low intracellular Zn increased DNA damage markers and induced p53 expression, but disrupted P53’s binding ability to its targets [85]. A similar effect was reported for NF-kB. As such, P53 elevation is likely a response to increased DNA damage in low-Zn conditions, but subsequent cellular responses (e.g., DNA repair) may be compromised due to defective protein activity. These suggest that P53 conformation and activation may be in such tight regulation by Zn that optimal levels are required for normal function.

Apoptosis due to low Zn levels seems to occur through the intrinsic pathway and can also be a manifestation of the direct effects of Zn on caspase activity [37,86]. The involvement of Zn deficiency in cell death was first observed due to its capacity to induce common morphological features of apoptosis, such as DNA and nuclear fragmentation, chromatin condensation, and formation of apoptotic bodies [86]. In 1992, Cohen and coworkers reported that Zn supplementation of thymocytes pretreated with the cell death inducer dexamethasone prevented the progression from peripheral nuclear chromatin condensation to nuclear collapse [87]. The effect was initially attributed to the suppression of the Ca^2+^/Mg^2+^-dependent endonuclease, an enzyme involved in DNA fragmentation and a sensitive target of Zn. However, it was then postulated that Zn might suppress biochemical reactions upstream of morphological events of apoptosis [86]. Researchers then reported inhibitory roles of Zn both for initiation and executioner caspases, supporting its antiapoptotic function [65,86]. Studies in several human epithelial cell lines (LIM1215, NCI-H292, A549) demonstrated that Zn chelation induced DNA fragmentation and apoptosis, by increasing caspase-3 and caspase-6 activation, while supplementation with exogenous Zn prevented the apoptogenic effects of the chelator [88]. The regulatory mechanisms of Zn may vary for each caspase. For example, caspase-8 and caspase-9, initiators of the extrinsic and intrinsic pathways, respectively, bind to two Zn ions at their active cysteine sites, which directly blocks substrate binding [35,65,89]. Furthermore, Zn also disrupts caspase-8 dimerization, an essential step for enzyme activity [6,89]. Activation of executioner caspase-3 from its zymogen precursor is also highly modulated by Zn, with low nanomolar concentrations being sufficient for enzyme inactivation [6,35,65,89]. Interestingly, first procaspase-activating compound (PAC-1), a proapoptotic protein required for procaspase-3 activation, operates by sequestering inhibitory Zn ions bound to the proenzyme, leading to caspase-3 autoactivation and apoptosis progression [89]. Another sensitive target for Zn is caspase-6 [6]. This protease cleaves pro-caspase-3 to its active form and is also responsible for lamin cleavage and dissolution of the nuclear membrane [82]. Nanomolar-to-micromolar concentrations of Zn are also able to inhibit caspase-6 and caspase-7 [6,35,65,82]. Altogether, Zn interactions with several caspases can limit the progression of apoptotic cascades by avoiding enzyme activation and proteolytic activity, ultimately downregulating apoptosis.

## 5. Zn and DNA Repair

Nutrigenomic studies show that a variety of enzymes related to DNA synthesis, damage sensing, repair, prevention of oxidative damage, and maintenance of DNA methylation patterns depend on micronutrients for proper functioning [90,91,92]. The participation of Zn in vital cellular processes and its requirement as a component of several DNA-interacting enzymes raised awareness of a possible role of this mineral as a modulator of DNA damage and repair capacity [93,94].

Zn is mostly known to support genome stability through several antioxidant mechanisms [6,43]. Firstly, as a component of copper (Cu)/Zn superoxide dismutase (SOD), Zn contributes to the removal of the superoxide anion (O_2_^•−^), a highly deleterious oxidant molecule [30,35,95]. Moreover, by competing with redox-active transition metals such as iron (Fe^2+^) and Cu^+^ for binding sites, Zn limits its ability to transfer electrons to H_2_O_2_ during Fenton reactions, thus reducing the formation of hydroxyl radicals (OH) [30,95,96]. Direct binding of Zn to the thiol and sulfhydryl groups, important sources of damage to biomolecules, also prevents protein oxidation [35,95,96]. Regulation of MT metabolism by Zn through MTF-1 also reduces oxidative stress [35,96,97]. Induced by increased Zn, MT are not only Zn buffers but also potent ROS scavengers [95,97]. Since the transcriptional regulation of MT is dependent on Zn, decreased MT expression has been observed in cellular models cultured with Zn deficiency and is associated with increased oxidative stress [97]. Lastly, another antioxidant mechanism of Zn is exerted through the Kelch-like ECH-associated protein 1 (KEAP1)–nuclear factor erythroid 2-related factor 2 (NRF2) complex (KEAP1–NRF2). NRF2 is a key cytoprotective transcription factor that regulates the expression of genes encoding antioxidant proteins and enzymes (e.g., GSH and SOD) [6,69]. NRF2 is normally bound to KEAP1, a Zn-binding protein that represses NRF2 activity. In oxidative stress conditions, multiple cysteine residues of KEAP1 are oxidized by ROS, causing structural changes followed by Zn release and complex dissociation [6,30]. Disruption of the KEAP1–NRF2 complex allows activation of NRF2 and its subsequent nuclear translocation, where the transcription factor binds to antioxidant-responsive elements (ARE) in the DNA promoter region of target genes to promote antioxidant responses and cytoprotection [6,30]. Thus, the increase in free Zn after KEAP1 oxidation is thought to serve chemopreventive purposes through NRF2 [6]. An experimental study of Zn depletion in mice shown an increase in oxidative stress markers associated with decreased NRF2 activity, accompanied by P53 activation and apoptosis [98]. Zn has also been reported to limit H_2_O_2_-induced damage to rat endothelial cells through NRF2-dependent stimulation of GSH biosynthesis, whereas Zn depletion decreased GSH levels and increased susceptibility of endothelial cells to oxidative stress [99]. In general, evidence suggests that Zn upregulates NRF2 activity to induce cytoprotective responses and manage oxidative stress. 

The many antioxidant properties of Zn maintain genome integrity by preventing oxidative lesions to DNA and other biomolecules (i.e., proteins and lipids) that can interact with DNA bases to form mutagenic adducts [35,95]. However, the roles of Zn in genome integrity are not limited to the prevention of oxidative stress. They are multilayered and regulate several DNA damage response (DDR) components directly involved in damage sensing and repair. Consequently, Zn is a cofactor and structural element of various enzymes involved in base excision repair (BER), nucleotide excision repair (NER), single-strand break (SSB) repair, and double-strand break (DSB) repair [95,100]. Additionally, key enzymes of the replication process are Zn metalloenzymes, such as DNA and RNA polymerase, TK, and replication protein A (RPA). Consequently, the inadequate availability of Zn affects several aspects of genome integrity, from DNA synthesis to cells’ responses to genotoxic stimuli and DNA repair capacity [82,96].

Studies under different Zn conditions indicate that a wide range of DDR-associated factors are differentially expressed in Zn-deficient cells compared to those in adequate Zn conditions. Such reports suggest that several components of the DNA repair machinery are induced by low Zn, most likely as a response to increased DNA damage, but their activity may be compromised by Zn deficiency. The most commonly reported effects of Zn deprivation on the genome are DNA oxidation, DNA strand breaks, and chromosomal lesions. For example, studies in rat models of Zn depletion have shown altered antioxidant status, elevated circulating oxidative stress markers, increased SSB in peripheral blood cells, and an increase in Zn-containing DNA repair proteins (i.e., 8-oxoguanine DNA glycosylase (OGG1) and P53) [101,102]. In rat glioma cells (C6 cell line), the elevated levels of ROS, reactive nitrogen species (RNS), and oxidative base lesions in Zn-deficient conditions were correlated with increased levels of apurinic/apyrimidinic endonuclease (APE) from BER, but also with a defective binding of NF-κB and AP1 transcription factors [85]. In normal prostate epithelial cells, Zn deficiency also increased SSB and differentially altered the expression of 286 genes, including genes related to DNA damage repair, cell cycle regulation, and apoptosis. Examples of such genes are *TP53*, *TP73*, *CCNT1*, *MRE11*, *XRCC4*, and *BRCA2*, all with overlapping functions in damage repair [97]. In contrast, moderate supplementation with Zn appears to reverse these damaging effects and alter the blood concentration of proteins involved in DNA repair, oxidative stress, and inflammation [103,104].

PARP, OGG1, APE, xeroderma pigmentosum complementation group A (XPA) protein, RPA, P53, and DNA ligase III are examples of DDR proteins that require Zn for adequate function. PARP1, a major sensor of SSB, has two specialized Zn fingers that recognize and bind to DNA lesions [105]. Once bound to DNA breaks, PARP1 promotes DNA repair by inducing post-translational modifications (i.e., PARylation) of the PARP1 protein itself and several targets [105]. This activity allows damage signaling and recruitment of repair factors to anchor in damaged sites. Zn depletion has been related to reduced PARP1 activity, which can be attributed to changes in its Zn content [101,106]. However, a critical role of PARP1 as a regulator of Zn deficiency-induced stress in neurons has also been stated, with PARylation of P53 increasing the stability and activity of this transcription factor [107]. 

BER bifunctional glycosylase OGG1 removes the mutagenic by-product of base oxidation 8-hydroxy-2′-deoxyguanosine (8-OHdG) and is also a Zn-finger protein. Recognition and binding to 8-OHdG occur through the Zn motif of OGG1. Moreover, OGG1 generates an intermediate SSB during base excision, requiring PARP1 cooperation for complete repair [51]. Studies of Zn depletion have shown an increase in 8-OHdG and higher levels of OGG1, but without changes in PARP1 expression, suggesting an altered response to damage in the absence of Zn [102,108]. APE2 from the BER pathway, which is involved in the repair of H_2_O_2_-induced lesions, also contains a Zn-finger domain involved in damage resection. Elevated levels of APE2 are found in several types of cancer, although they are not always functional [95]. Concomitantly with the low-Zn status, this increase may most likely be a response to increased oxidative damage induced by Zn deficiency [85].

The protein XPA, key in the NER pathway, also has a Zn-finger motif [30,109]. XPA does not display catalytic activity, but its Zn finger is required to recognize helix-distorting lesions (e.g., those induced by ultraviolet (UV) radiation and chemotherapeutic agents) and recruit and stabilize repair factors [30]. For example, RPA is recruited to repair sites to stabilize the undamaged strand, and its activity requires changes in the redox potential within the cell, which is only possible due to one of its subunits with a Zn-finger motif [30,109]. This motif is essential for optimal DNA-binding ability and enzyme function [109].

The requirement of Zn for the function of P53 is of primary importance in the DDR. Coordination of cell cycle, apoptosis, and repair responses is essential for an adequate damage response. Upon DNA damage, P53 induces cell cycle arrest to provide time for damage repair before cytokinesis is completed [110]. Additionally, P53 induces the expression of repair genes or triggers apoptosis or senescence in case of irreparable damage [110]. Impairment of P53 function, commonly reported under low-Zn conditions, poses significant threats to genome homeostasis, as dysfunctional P53 cannot efficiently eliminate DNA damage. In normal prostate epithelial cells, Zn deficiency increased P53 nuclear expression, but compromised its DNA-binding ability, resulting in the downregulation of P53 target genes [97]. Similar findings have been reported in other cell types, such as human lung fibroblasts [108]. These examples demonstrate how several interconnected proteins depend on Zn to maintain genome integrity and how insufficient Zn can significantly limit DDR.

Other trace elements display functions similar to Zn in relation to oxidative stress and DNA damage prevention. Selenium (Se), for example, is essential for genome integrity, mainly due to its potent antioxidant function [110]. Zn-dependent repair factors, such as BER glycosylases and P53, are also influenced by Se. P53-induced repair responses after genotoxic exposure are enhanced by cellular pretreatment with Se. However, the combined effects of Zn and Se on DNA repair factors may be detrimental, depending on the Se dose and different forms [110]. Reducible Se compounds can negatively influence Zn-dependent DDR processes through oxidation of the Zn-finger motifs from DNA repair proteins. Such interactions promote Zn release and inhibit DNA repair [110]. Therefore, when Se disrupts Zn homeostasis, the cytoprotective functions of Zn may be lost. Therefore, considering the interactions of possible micronutrients and their variable outcomes, an adequate balance between trace elements seems crucial in maintaining adequate repair responses. Table 1 summarizes the main mechanisms targeted by Zn with known implications for maintaining genome integrity. 

## 6. The Therapeutic Potential of Zn

Zn and Zn transporters are involved in the pathophysiology of many human conditions, ranging from cancer to neurological and neuropsychiatric disorders, aging, diabetes, and infection [13,35].

The relationship between Zn deficiency and the development of several malignant neoplasms has been established in recent years, and the antitumor efficacy of Zn has been demonstrated in several types of cancer [111]. Considering the broad spectrum of Zn functions (i.e., modulation of cell signaling, proliferation, apoptosis, inflammation, DDR, and antioxidant defenses), altered Zn homeostasis can drive carcinogenesis in a multilayered way [111,112,113]. Most patients with lung, breast, liver, stomach, prostate, and head and neck cancers consistently present dysregulated Zn levels [111,113,114,115,116,117]. These changes are often accompanied by altered ZnT and ZIP expression and are considered adaptive strategies of cancer cells to avoid the cytoprotective effects of Zn and increase mutagenesis [111]. The downregulation of ZIP1, ZIP2, and ZIP3, for example, has been linked to Zn deficiency in prostate cancer [118,119]. Decreased expression of ZnT4 has also been related to progression from early disease to invasive prostate cancer disease [120]. Zn depletion during hepatocellular carcinoma development and progression has been attributed, in part, to a downregulation of ZIP14, while the increased expression of ZIP10 has been related to a more invasive phenotype and lymph node metastasis in breast cancer [121,122]. Changes in ZIP3 expression have also been associated with the development of pancreatic adenocarcinoma [123]. Altogether, it is suggested that changes in Zn status allows cancer cells to acquire a set of biological advantages that allow malignant transformation. These observations may raise opportunities for Zn-based anticancer therapies. Kocdor and colleagues, for example, have shown that Zn supplementation resulted in significant growth inhibition, increased apoptosis, and sensitization to docetaxel treatment in a model of non-small-cell lung cancer [124]. A study by Zhang et al. also found that Zn improved chemosensitivity to paclitaxel in the prostate cancer cell line PC-3 [125]. A recent study by our group also showed that Zn supplementation differentially modulates the genotoxicity of DNA-damaging agents depending on the cell context [126]. Specifically, we found that normal human lymphocytes supplemented with Zn sulfate (ZnSO_4_) showed lower scores of chromosomal damage biomarkers after genotoxic exposure compared to lymphocytes from standard cell culture conditions and Zn depletion cultures. Moreover, repair kinetics following genotoxic stimuli was also improved in Zn-supplemented cells. In contrast, we observed that supplementing an acute myeloid leukemia cell line (HEL) with ZnSO_4_ increased the genotoxicity of H_2_O_2_ and UVC radiation, impaired repair kinetics, and led to the persistence of damage. These results point to a cytoprotective effect against DNA damage sources in normal cells and a genotoxic effect in malignant cells, which can be exploited for therapeutic purposes. Other authors have previously indicated that the cytoprotective effects of Zn may not manifest in malignant cells. Wysokinski and colleagues reported that Zn potentiated the genotoxicity of anthracyclines in acute lymphoblastic leukemia cells and prevented it in normal lymphocytes [127]. Sliwinski et al. found similar results after exposure of a chronic myeloid leukemia cell line to H_2_O_2_ [128].

Taken together, these data open possibilities for Zn supplementation strategies to improve anticancer treatment by applying lower-dose, but equally successful, therapeutic regimens and minimizing side effects. However, detailed studies of the effects of Zn in different cancer subtypes and disease stages are required to address the adequacy of Zn supplementation in cancer patients fully. It is possible that, similar to other biomolecules and compounds, Zn shows cell type-, concentration-, and disease stage-dependent effects, which makes it mandatory to utilize appropriate supplementation strategies for specific contexts rather than making absolute recommendations [111]. Although Zn deficiency is commonly found in early stages of disease and is assumed to play a prominent role in tumor initiation and development, some established neoplasms also show ZIP upregulation and increased intracellular Zn levels [35,111]. A notable exception is the case of prostate cancer, which consistently presents considerably lower intracellular Zn than normal prostate cells [30,35,111,113]. Several in vitro and in vivo studies in prostate cancer models indicate antitumoral effects of Zn supplementation, mainly due to antiproliferative, proapoptotic, and invasion and migration suppression functions [129,130,131,132,133,134]. ZnR/GPR39 is also overexpressed in several neoplasms. Experiments in the human breast cancer cell line MCF-7, for instance, revealed that ZnR/GPR39 is active in breast cancer cells, and activates MAPK/ERK and PI3K/AKT signaling pathways, resulting in increased proliferation and invasiveness [135]. This receptor may provide an interesting target for therapeutic tools, with receptor silencing presenting itself as a possible approach to reduce tumorigenicity in cancers with high receptor activity [12].

Zn also plays an important role in cardiovascular diseases. The data show a strong association between reduced Zn and several cardiovascular conditions (e.g., ischemic cardiomyopathy, atrial fibrillation, congestive heart failure, and coronary heart disease) [27,35]. Beneficial applications of Zn against myocardial ischemia–reperfusion injury have been reported in rodent models, improving myocardial recovery and decreasing arrhythmias [27,35]. The importer ZIP7 has also been implicated in the control of myocardial reperfusion injury, by regulation of mitophagy [136]. Changes in expression of other Zn transporters (e.g., ZIP8) and the ZnT/GPR39 receptor in endothelial and vascular smooth muscle cells have also been implicated in important driver events of altered Zn homeostasis and cardiovascular disease [137,138]. Altered expression of ZIP12 has also been associated with pulmonary hypertension [139].

Neurological disorders are also related to aberrant Zn regulation. Zn deprivation is associated with altered behavior, depression, decreased cognitive function, and increased predisposition to epileptic seizures [12,30,35,140]. Animal and human studies indicate that decreased serum Zn enhances depression-like behaviors. Changes in ZnR/GPR39 expression levels arising from Zn deficiency have also been observed in studies of depression and are related to behavioral manifestations; dietary interventions with Zn successfully alleviated symptoms [12,35,141,142,143]. Schizophrenia has also been associated with altered expression of ZIP8 [138,144]. Changes in serum Zn have also been reported in epilepsy patients, and some interventions regarding Zn supplementations have yielded positive results in epilepsy treatment [140]. These results have been attributed to the varied mechanisms of Zn regulation in the nervous system, such as modulation of the receptors NDMA, GABA, and ZnR/GPR39, and changes in Zn transporters and hormonal regulation [12,30,35,140,141]. A decrease in Zn uptake and usage also increases the susceptibility of senescent cells to apoptosis and increases participation in neurodegenerative conditions [141]. Changes in the expression of ZIP8 and ZIP14 have been found in Parkinson’s disease [138]. Familiar forms of amyotrophic lateral sclerosis commonly present with a SOD mutation that leads to a decreased affinity for Zn and toxic gain of function in motor neurons, suggesting that disturbed Zn homeostasis also contributes to this condition [141]. In the case of Alzheimer’s disease, an abnormal enrichment of Zn in β-amyloid plaques is commonly found in the postmortem examination of patients’ brains [35,141,145]. Studies suggest that Zn is involved in the precipitation of β-amyloid and its aggregation into senile plaques, the main hallmarks of the disease [35,141,145,146,147]. Since Zn chelation has been shown to limit the formation of these plaques, modulating Zn status has been pointed out as a potential therapeutic strategy for Alzheimer’s disease [35,145].

The normal development and function of the immune system also require Zn [21,35,112]. Many aspects of the immune response are Zn-dependent, such as granulocyte recruitment, cytokine secretion, and cytotoxicity [18,52,68]. Furthermore, Zn exerts a powerful influence on T lymphocytes, and Zn deficiency is related to thymus atrophy, altered lymphocyte maturation, and lymphopenia [11,21,30,112,148]. Not surprisingly, a decline in Zn is related to immune dysfunction, compromised cell- and antibody-mediated immune responses, and increased incidence of infections [11,21,30,112]. Positive results of Zn supplementation have been observed in the management of symptoms of common cold, parasitic, viral, and bacterial infections [21,30,96]. Since Zn regulates the responses in mast cells, Zn imbalances are also associated with the development of allergic diseases, which can be attenuated by increased dietary or supplementary Zn [31,149]. The reduction in the frequency, duration, and severity of acute diarrhea in malnourished children with Zn supplementation has also been extensively reported [14,150]. This effect is supported by the involvement of Zn transporters and the ZnR/GPR39 receptor in the regulation of ion transport mechanisms, the formation of tight junctions, the maintenance of gut barrier permeability, and immunocompetence [14,112]. The regulatory role of Zn in intestinal barrier permeability has been implicated in attenuating inflammatory bowel diseases such as Crohn’s disease and ulcerative colitis [14]. 

A well-known therapeutic application of Zn concerns dermatology. There is an established association between skin conditions and Zn deficiency and/or disruption of Zn transporters [14,151]. Several genetic disorders with skin manifestations (e.g., atopic dermatitis, enteropathic acrodermatitis, dysplastic form of spondylocheiro of Ehlers–Danlos syndrome, and epidermodysplasia verruciformis) are caused by mutations and/or dysregulation of Zn transporters such as ZIP10, ZIP4, ZIP13, and ZnT1 [138,151]. Furthermore, the nutritional deficiency of Zn also results in skin problems, which can be partially or entirely solved by supplementation [151]. The topical application of Zn lotions is also commonly prescribed to accelerate wound healing and re-epithelialization due to ZnR/GPR39-dependent stimulation of proliferation, migration, and anti-inflammatory pathways, particularly in keratinocytes and skin fibroblasts [12,13,14].

Lastly, a role for Zn in diabetes is also known. Pancreatic β-cells contain a notable amount of Zn, which is required for cells’ functional regulation [12,112,137,152]. Accordingly, Zn is fundamental for insulin synthesis, storage, and structural stability [137]. Furthermore, the concomitant release of Zn and insulin from pancreatic β-cells is important for adequate insulin clearance [152]. Pancreatic β-cell dysfunction and insulin resistance underlie the onset of type 2 diabetes (T2D), while in type 1 diabetes (T1D), the immune system attacks pancreatic β-cells limiting insulin production. Decreases in intracellular Zn have been observed in both animal models and patients with T1D and T2D, suggesting that Zn contributes to both types of diabetes [12,112,137,152]. The Zn transporter ZnT8, involved in the accumulation of Zn in the insulin secretory granule, plays a particularly important role in these imbalances and may even have diagnostic value in diabetes [153]. Mutations in the *ZNT8* gene have been implicated in T2D, and the variant Arg325 *ZNT8* is related to T2D and autoimmune T1D [12,112,152]. Prospective studies have shown that Zn deficiency and ZnT8 dysfunctions increase the risk of glucose intolerance, suggesting that adequate Zn status and Zn transporter function are critical aspects of preventing glucose homeostasis disruption and the onset of disease [137,152]. ZIP13 expression also appears to be dysregulated in T2D [138]. Thus, accessing Zn status may be a valuable tool for disease prediction and correcting Zn deficiencies can support therapeutic approaches in diabetes. Table 2 indicates the known implications of several ZIP and ZnT in human diseases.

## 7. Conclusions

**Figure 5:**
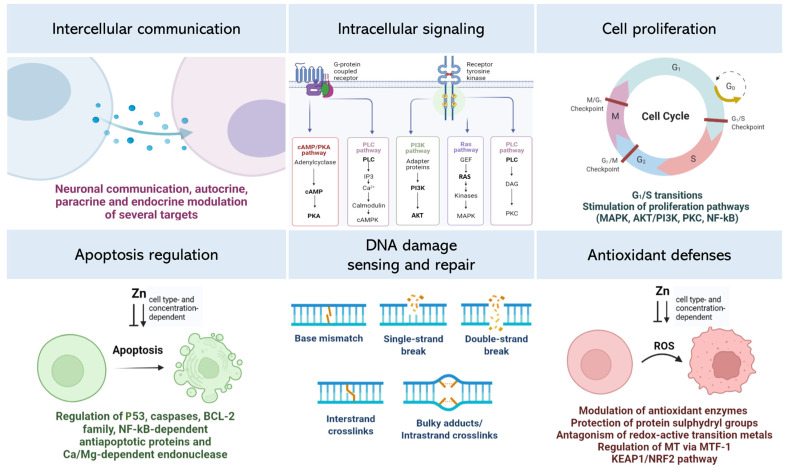
Biological roles of Zn. Zn is a vital trace element and displays various fundamental biological functions. Zn mediates extracellular communication by interacting with several receptors in different cell types. It also acts intracellularly, modulating key signaling pathways. The regulation of cell proliferation and apoptosis also requires Zn. The DNA damage response (DDR) mechanisms are also Zn-dependent since several damage-sensing and repair proteins use Zn for structural, catalytic, and regulatory functions. The role of Zn as an antioxidant element and regulator of cell antioxidant defenses is also known. (Figure created with Biorender)

Zn is a vital micronutrient in the human body. As components of numerous enzymes and transcription factors, Zn ions regulate the activity of various biomolecules involved in many biological mechanisms (Figure 5). Moreover, Zn itself acts as a signaling factor in key cellular pathways, and Zn status is dynamically altered to promote different cellular responses. 

The roles of Zn in the regulation of DNA synthesis, cell growth, proliferation, survival, and apoptosis are well-documented and supported by a significant scientific research background. However, some Zn targets and mechanisms of regulation still need further characterization. Another remarkable aspect of Zn biology lies in its antioxidant effects, which are key in preventing oxidative damage to biomolecules, cells, and tissues. Furthermore, increasing evidence supports the involvement of Zn in modulating DNA damage and cell repair responses because of its role as a cofactor of several enzymes implicated in DDR reactions. 

Notwithstanding the remarkable effects of Zn in cell biology, the wide range of Zn effects may be largely dependent on Zn concentration and cell type and may even differ considerably under normal and pathological conditions. Thus, the overall impact of this multifunctional trace element requires dose-, cell type-, and context-dependent considerations. Imbalances in Zn homeostasis and Zn bioavailability due to malnutrition and altered expression of Zn transporters are related to the pathophysiology of several human disorders. Although some pathological events are related to increased Zn status, more importance is given to Zn deficiency as a disease inducer, rendering the study of Zn supplementation strategies for different human conditions a very pertinent topic for future biomedical research.

## Figures and Tables

**Table 1:** Zinc (Zn) targets with implications for the maintenance of genome stability.

Cellular Mechanisms	Zn Implication	Biological Effect
Antioxidant defenses	Cu/Zn SOD	Decreasing oxidative stress and oxidative DNA lesions
	Fenton reactions	
	Binding to thiol and sulfhydryl groups	
	ROS buffering by MT	
	KEAP1-NRF2	
Replication	DNA and RNA polymerases	Cell cycle regulation
	TK	
	RPA	
Tumor suppression	P53	Cell cycle and apoptosis regulation
DDR	PARP1	BER and SSB repair pathways
	OGG1	
	APE2	
	XPA	NER pathway

**Table 2:** Zn transporters and their implications in human diseases.

Zn Transporters	Disease Implications	References
ZIP1	Alzheimer’s disease	[111,112,119,140,145]
	Pancreatic cancer	
	Prostate cancer	
	Seizures	
ZIP2	Cardiovascular diseases	[111,112,118,137]
	Pancreatic cancer	
	Prostate cancer	
ZIP3	Pancreatic cancer	[111,112,115,118,123,140]
	Prostate cancer	
	Seizures	
ZIP4	Acrodermatitis enteropathica	[111,112,137,138,151]
	Cancer cachexia	
	Hepatocellular carcinoma	
	Pancreatic cancer	
ZIP6	Breast cancer	[30,111,138]
	Esophageal squamous cell carcinoma	
ZIP7	Breast cancer	[30,111,136,138]
	Gastric cancer	
ZIP8	Cardiovascular diseases	[137,138,144]
	Chron’s disease	
	Parkinson’s disease	
	Schizophrenia	
ZIP9	Breast cancer	[111]
ZIP10	Atopic dermatitis	[30,111,121,138,151]
	Breast cancer	
	Sepsis	
ZIP12	Cardiovascular diseases	[137,138,139]
	Pulmonary hypertension	
ZIP13	Ehlers–Danlos syndrome (Spondylocheiro dysplastic form)	[137,138,151]
	Type 2 diabetes	
ZIP14	Parkinson’s disease	[111,138]
	Cancer cachexia	
ZnT1	Atrial fibrillation	[35,138,145,151]
	Alzheimer’s disease	
	Epidermodysplasia verruciformis	
ZnT3	Alzheimer’s disease	[12,35,140,145]
	Seizures	
	Behavioral and cognitive impairment	
ZnT4	Prostate cancer	[111,120]
ZnT6	Alzheimer’s disease	[145]
ZnT7	Alzheimer’s disease	[12,35]
	Prostate cancer	
ZnT8	Autoimmune type 1 diabetes	[12,35,112,137,152,153]
	Type 2 diabetes	

ZIP, Zrt- and Irt-like proteins; ZnT, zinc transporters.

## Data Availability

Not applicable.

## Abbreviations

8-OHdG, 8-hydroxy-2′-deoxyguanosine; AIF, apoptosis-inducing factor; AKT, protein kinase B; APE, apurinic/apyrimidinic endonuclease; ARE, antioxidant-responsive elements; BAD, BCL-2-associated death promoter; BAX, BCL-2-associated X protein; BCL-2, B-cell leukemia/lymphoma 2 protein; BCL-xL, B-cell lymphoma-extra-large protein; BER, base excision repair; Ca^2+^, calcium; cAMP, cyclic adenosine monophosphate; CaMPK-2, Ca^2+^/calmodulin-activated protein kinase-2; CDK, cyclin-dependent kinases; cGMP, cyclic guanosine monophosphate; c-IAP1, cellular inhibitor of apoptosis protein 1; Cl^-^, chlorine; Cu, copper; Cu/Zn SOD, copper–zinc superoxide dismutase; DDR, DNA damage response; DSB, double-strand breaks; EGF, epidermal growth factor, ER, endoplasmic reticulum; ERK, extracellular signal-regulated kinases; Fe^2+^, iron; GABA, gamma-aminobutyric acid; GH, growth hormone; GSH, glutathione; H^+^, hydrogen; H_2_O_2_, hydrogen peroxide; IGF, insulin growth factor; IL-1β, interleukin-1 beta; IP3, inositol triphosphate; JAK2, Janus kinase 2; K^+^, potassium; KEAP1, Kelch-like ECH-associated protein 1; LPS, lipopolysaccharides; MAPK, mitogen-activated protein kinase; MRE11, double-strand break repair protein MRE11; MT, metallothioneins; MTF-1, metal response element-binding transcription factor-1; Na^+^, sodium; NER, nucleotide excision repair; NF-kB, nuclear factor kappa-light-chain-enhancer of activated B cells; NMDA, N-methyl-D-aspartate; NO, nitric oxide; NRF2, nuclear factor erythroid 2-related factor 2; O_2_^•−^, superoxide anion; OGG1, 8-oxoguanine DNA glycosylase; ^•^OH, hydroxyl radical; P53, tumor protein 53; P70S6K, P70S6 kinase; P73, tumor protein 73; PAC-1, first procaspase-activating compound; PARP, Poly(ADP-ribose) polymerase; PDE, cyclic nucleotide phosphodiesterases; PI3K, phosphoinositide 3-kinase; PKC, protein kinase C; PTP, protein tyrosine phosphatases; RNS, reactive nitrogen species; ROS, reactive oxygen species; RPA, replication protein A; Se, selenium; SOD, superoxide dismutase; SSB, single-strand breaks; STAT3, signal transducer and activator of transcription 3; T1D, type 1 diabetes; T2D, type 2 diabetes; TK, thymidine kinase; TNF-α, tumor necrosis factor-alpha; TRPA1, transient receptor potential ankyrin 1; UV, ultraviolet; VGCCs, voltage-gated calcium channels; XIAP, X-linked inhibitor of apoptosis protein; XPA, xeroderma pigmentosum complementation group A; XRCC4, X-ray repair cross-complementing protein 4; ZIP, Zrt- and Irt-like proteins; Zn, zinc; ZnR/GPR39, G protein-coupled Zn-sensing receptor ZnR/GPR39; ZnSO_4_, zinc sulfate; ZnT, zinc transporters.
